# Interaction of Tau with the chemokine receptor, CX3CR1 and its effect on microglial activation, migration and proliferation

**DOI:** 10.1186/s13578-020-00474-4

**Published:** 2020-09-15

**Authors:** Hariharakrishnan Chidambaram, Rashmi Das, Subashchandrabose Chinnathambi

**Affiliations:** 1 Neurobiology Group, Division of Biochemical Sciences, CSIR-National Chemical Laboratory, Dr. Homi Bhabha Road, 411008 Pune, India; 2 Academy of Scientific and Innovative Research (AcSIR), 411008 Pune, India

**Keywords:** Alzheimer’s disease, Microglia, Neuron, Tau, Fractalkine, CX3CR1 receptor

## Abstract

Alzheimer’s disease (AD) is a neurodegenerative disease that leads to progressive loss of memory and dementia. The pathological hallmarks of AD include extracellular accumulation of amyloid-β peptides forming senile plaques and intracellular accumulation of Tau oligomers and filamentous species. Tau is a microtubule-binding protein that stabilizes tubulin to form microtubules under physiological condition. In AD/ pathological condition, Tau detaches from microtubules and aggregates to form oligomers of different sizes and filamentous species such as paired helical filaments. Microglia are the resident brain macrophages that are involved in the phagocytosis of microbes, cellular debris, misfolded and aggregated proteins. Chemokine receptor, CX3CR1 is mostly expressed on microglia and is involved in maintaining the microglia in a quiescent state by binding to its ligand, fractalkine (CX3CL1), which is expressed in neurons as both soluble or membrane-bound state. Hence, under physiological conditions, the CX3CR1/CX3CL1 axis plays a significant role in maintaining the central nervous system (CNS) homeostasis. Further, CX3CR1/CX3CL1 signalling is involved in the synthesis of anti-inflammatory cytokines and also has a significant role in cytoskeletal rearrangement, migration, apoptosis and proliferation. In AD brain, the expression level of fractalkine is reduced, and hence Tau competes to interact with its receptor, CX3CR1. In microglia, phagocytosis and internalization of extracellular Tau species occurs in the presence of a chemokine receptor, CX3CR1 which binds directly to Tau and promotes its internalization. In this review, the pathophysiological roles of CX3CR1/fractalkine signalling in microglia and neurons at different stages of Alzheimer’s disease and the possible role of CX3CR1/Tau signalling has been widely discussed.

## Background

Alzheimer’s disease is a neurodegenerative disease that leads to progressive loss of memory and dementia [1]. Alzheimer’s disease mostly occurs at the age of 65 and above, even though age is not the only factor for this disease [2]. Several other factors such as genetic and environmental factors (eg. stress) also play a critical role in the onset of Alzheimer’s disease [3, 4]. Extracellular deposition of senile plaques from amyloid-β (Aβ) peptides and intracellular accumulation of Tau oligomers and aggregates are the pathological features of Alzheimer’s disease [5, 6]. Amyloid precursor protein (APP) is a single transmembrane protein which is predominantly expressed in the central nervous system. Under physiological conditions, non-amyloidogenic APP processing occurs, which involves APP cleavage by α- and γ-secretases yielding soluble-APPα (sAPPα), APP intracellular domain (AICD) and p3 [7]. sAPPα secreted from cleavage of APP is involved in neuroprotection and neurite outgrowth [8, 9]. In AD, an imbalance occurs in the proteolysis of APP towards amyloidogenic processing that leads to the secretion of Aβ. The levels of α-secretase had been reduced, which alters the proteolytic cleavage of APP by β- and γ-secretases yielding sAPPβ, AICD and Aβ [7, 10]. Proteolytic cleavage of APP by α- and β-secretases are regulated by several G-protein coupled receptors (GPCRs) that leads to secretion and accumulation of Aβ plaques under pathological conditions [7]. Tau is a microtubule-binding protein that stabilizes tubulin to form microtubules in the neuronal cells [11, 12]. In humans, Tau gene is located on chromosome 17q21. Tau is predominantly expressed in the axons of matured neurons and encodes for six different isoforms. Full-length Tau consists of 441 amino acids with an N-terminal domain, two N-terminal inserts (N1 and N2), two proline-rich domains (PRD1 and PRD2), four repeat regions that form microtubule-binding domain (R1-R4) and a C-terminal domain. The isoforms are generated based on the alternative splicing of exons that encodes for N1, N2 inserts and R2 region of repeat domain to form 2N4R (hTau40), 1N4R (hTau 34), 0N4R (hTau 24), 2N3R (hTau 39), 1N3R (hTau 37) and 0N3R (hTau 23). Tau is a phosphoprotein that requires basal phosphorylation for its physiological functioning. In Alzheimer’s disease, Tau undergoes several post-translational modifications such as hyperphosphorylation, glycation, glycosylation, nitration, methylation, sumoylation, truncation, etc*.,* [13]. Among the various post-translational modifications, Tau phosphorylation is widely studied and majorly contributes to Tau pathology that leads to its detachment from microtubules, misfolding and intracellular accumulation to form oligomeric and filamentous species [13, 14]. Currently, Tau-based therapy is attaining more attention over Aβ-based treatment and this led to the discovery of various mechanisms for inhibiting Tau-mediated pathology such as inhibition and reversal of post-translational modifications, aggregation inhibition and disaggregation of preformed fibrils [15–23].

## G-protein coupled receptors in Alzheimer’s disease

GPCRs belong to the largest class of membrane proteins that are involved in cell-to-cell communications, signal transduction pathways and hormonal regulation [24, 25]. The human genome contains about 800 different GPCRs that are classified as five different classes (rhodopsin, secretin, glutamate, adhesion, and frizzled/taste receptors) [26]. GPCRs contain seven transmembrane domains made of α-helices with an extracellular N-terminal and intracellular C-terminal domain [27]. GPCRs respond to a variety of extracellular signals such as hormones, neurotransmitters, proteins, peptides, lipids, ions, photons, etc., by a change in structural conformation [28]. Upon GPCR activation, the bound G-proteins are activated by exchanging the GDP with a GTP and initiate the downstream signalling cascades such as activation/inhibition of secondary signalling molecules, cellular kinases, nuclear transcription factors, etc., [29] GPCR signalling is also mediated by β-arrestin, a G-protein independent pathway that involves phosphorylation of membrane GPCRs by GPCR kinases (GRKs), followed by binding of β-arrestin in the intracellular region [30]. β-arrestins are the scaffolding proteins that promote receptor desensitizing, internalization and activation of several other intracellular signals [31]. There are several other mechanisms of GPCR activation such as intracellular activation, transactivation, dimerization activation, biphasic activation and biased activation [32]. GPCRs are the major drug targets, and approximately 700 approved drugs targeting GPCRs accounts for 35% of approved drug targets [33]. In AD, the pathophysiological role of GPCRs has been widely studied, and there are several reports stating that GPCRs are playing a significant role in both amyloid-β and Tau hypothesis [7, 34, 35]. The cDNA microarray analysis obtained from AD patients also stated that the expression of several GPCRs had been altered when compared with controls [36]. Several neuronal GPCRs such as A1 adenosine receptor, Angiotensin II type-2 receptor, Metabotropic glutamate receptor-2, CXCR2 and CCR3 chemokine receptors are involved in Tau phosphorylation mediated by several cellular kinases such as GSK-3β, ERKs and CDK5 kinases [34], whereas GPCRs such as M1 muscarinic acetylcholine receptor, β2-adrenergic receptor and corticotropin releasing factor receptor-1 are involved in downregulation of Tau phosphorylation (briefly reviewed by Chidambaram et al. 2020) [34]. The neuronal GPCRs, muscarinic acetyl choline receptors were been hypothesized to interact with extracellular Tau resulting in the increased level of intracellular calcium on Tau exposure to neuroblastoma cells [37]. Later, identified that Tau activates M1 and M3 muscarinic acetylcholine receptors by its interaction and promotes intracellular calcium levels in neuronal cells. [37–39]. In addition, CX3CR1 chemokine receptor in microglia acts as surface receptor for extracellular Tau that interacts and promotes its internalization [40]. These evidences highlight the pathophysiological role of GPCRs in Tauopathy.

## Microglia—scavengers of misfolded proteins

Microglia are the resident macrophages that act as innate immune cells of the brain and play a significant role in regulating brain development [41, 42]. Microglia are derived from primitive yolk sac progenitors and contributes to around 10–15% of total brain cells [43, 44]. Microglia are capable of changing the morphology in response to their microenvironment [45]. Microglia are primarily involved in constant surveillance of its microenvironment, phagocytosis of microbes, cellular debris, damaged cells and aggregated proteins [46]. Microglia are also involved in synaptic pruning, *i.e*. phagocytosis and clearance of excessive neuronal cells and synapse leading to proper neuronal function and synaptic plasticity in developing CNS [47]. In healthy condition, microglia are maintained at a quiescent stage by several intrinsic (Runt-related transcription factor-1, Interferon regulatory factor-8, etc.,) and extrinsic factors (CD200, CX3CR1, TREM2, etc*.,*) [48]. Several cytokines and neurotransmitters released by neurons either as ‘secreted’ or ‘membrane-bound’ are involved in the neuron-glia cross talk to maintain microglial homeostasis [49, 50]. Microglia are activated in response to a variety of factors present in their microenvironment. Microglial receptors such as toll-like receptors, Triggering receptor expressed on myeloid cells 2 (TREM-2), etc., receives the signals from the environment and activates microglia in response to infection or inflammation [51–53]. The cytokines from type-1 T-helper cells such as IFN-γ and lipopolysaccharides are involved in pro-inflammatory activation of microglia (M1-microglia) and the cytokines from type-2 T-helper cells and dietary fatty acids are involved in the anti-inflammatory activation of microglia (M2-microglia) [44, 52–55]. In Alzheimer’s disease, microglia have both beneficial as well as harmful effects depending on disease stage and progression. There are several reports stating that microglia are primarily involved in phagocytosis and clearance of plaques, fibrils and damaged cells [56, 57]. Microglial priming and presenting self-antigen such as Tau and amyloid-β to the infiltrated CD4 + T lymphocyte cells through MHC I/II molecules alter its phenotype from M1 to form M2 stage which is involved in phagocytosis, tissue remodelling and neuroprotection [58]. Genome-wide association studies reported over 20 genetic loci that are related to AD, among which several genes are selectively expressed in microglia [59, 60]. TREM2 is one such receptor that plays a pivotal role in phagocytosis and clearance of apoptotic neurons, bacteria, lipoproteins, etc., [61–63]. Mutations in the TREM2 gene (R47H) increases the risk of AD that leads to impaired microglial activation and phagocytosis [64]. In microglia, there are various receptors involved in receptor-mediated endocytosis of amyloid-β. These include scavenger receptors, toll-like receptors, transmembrane receptors such as integrin and Triggering receptor expressed on myeloid cells 2 reported to interact directly with amyloid-β fibrils for its activation, phagocytosis and internalization [35, 65, 66]. In AD, extracellular Tau species activates microglia via actin remodelling, promotes migration and phagocytosis of Tau for its clearance which could further be enhanced by other factors such as lipids and fatty acids [67–69]. In Tauopathy models, microglia are involved in Tau propagation, neuroinflammation and neuronal damage by the release of toxic factors and pro-inflammatory cytokines and also leads to synaptic losses [70, 71]. Extracellular Tau treatment activates p38 MAPK and alters the pathway to express pro-inflammatory cytokines in primary microglia cells [72]. The gene expression of pro-inflammatory cytokines such as IL-6, IL-1β, TNF-α and Mip-1α has been increased in the microglial cells upon exposure to extracellular Tau [72]. Though there are not many studies performed on microglial surface receptors for Tau interaction and internalization, recent findings report that the chemokine receptor, CX3CR1 promotes Tau phagocytosis and internalization [40].

## CX3CR1 receptor—physiological functions in the CNS

CX3CR1 is a chemokine receptor that belongs to the class A family of G-protein coupled receptors. In humans, CX3CR1 is abundantly expressed in white blood cells such as lymphocytes and monocytes for their migration [73]. In the CNS, CX3CR1 receptor is largely expressed by microglial cells (brain macrophages) followed by neuronal cells that are involved in cell adhesion and migration [74–77]. Fractalkine (CX3CL1), the ligand for this receptor is constitutively expressed by neuronal cells and to a lesser extent by astrocytes and are present as either membrane-bound or soluble form [78, 79]. Fractalkine consists of total 373 amino acids among which the N-terminal domain and the mucin-like stalk forms the extracellular flanking residues. The N-terminal domain constitutes 76 amino acid residues, mucin-like stalk constitutes 241 amino acid residues, the transmembrane region constitutes 19 amino acid residues, and the intracellular C-terminal region constitutes 37 amino acid residues [80]. The N-terminal peptide and the mucin-like stalk of the membrane-bound protein are cleaved by many proteolytic enzymes such as cysteine proteases, cathepsin-S, and metalloproteinases such as disintegrin and metalloproteinase domain-containing protein 10 (ADAM10), disintegrin and metalloproteinase domain-containing protein 17 (ADAM17) to form the soluble fractalkine [81–83]. The membrane-bound fractalkine acts as an adherent and maintains the microglia in resting state, whereas the soluble fractalkine acts as a chemoattractant promoting microglial migration towards the site of infection or inflammation [84]. This leads to the neuron-microglia cross talk in the development of brain such as learning, memory and synaptic plasticity [85]. CX3CR1 is a G_i_ protein-bound GPCR that on fractalkine binding activates several signalling molecules and transcription factors such as nuclear factor kappa-light-chain-enhancer of activated B cells (NFκB) and cAMP response element-binding protein (CREB) [85, 86]. The activated transcription factors are involved in regulating microglial activation, cytokine synthesis, migration, proliferation and neuronal survival. [77, 85, 86].

## CX3CR1/Fractalkine signalling—different roles in Microglia

**Fig. 1:**
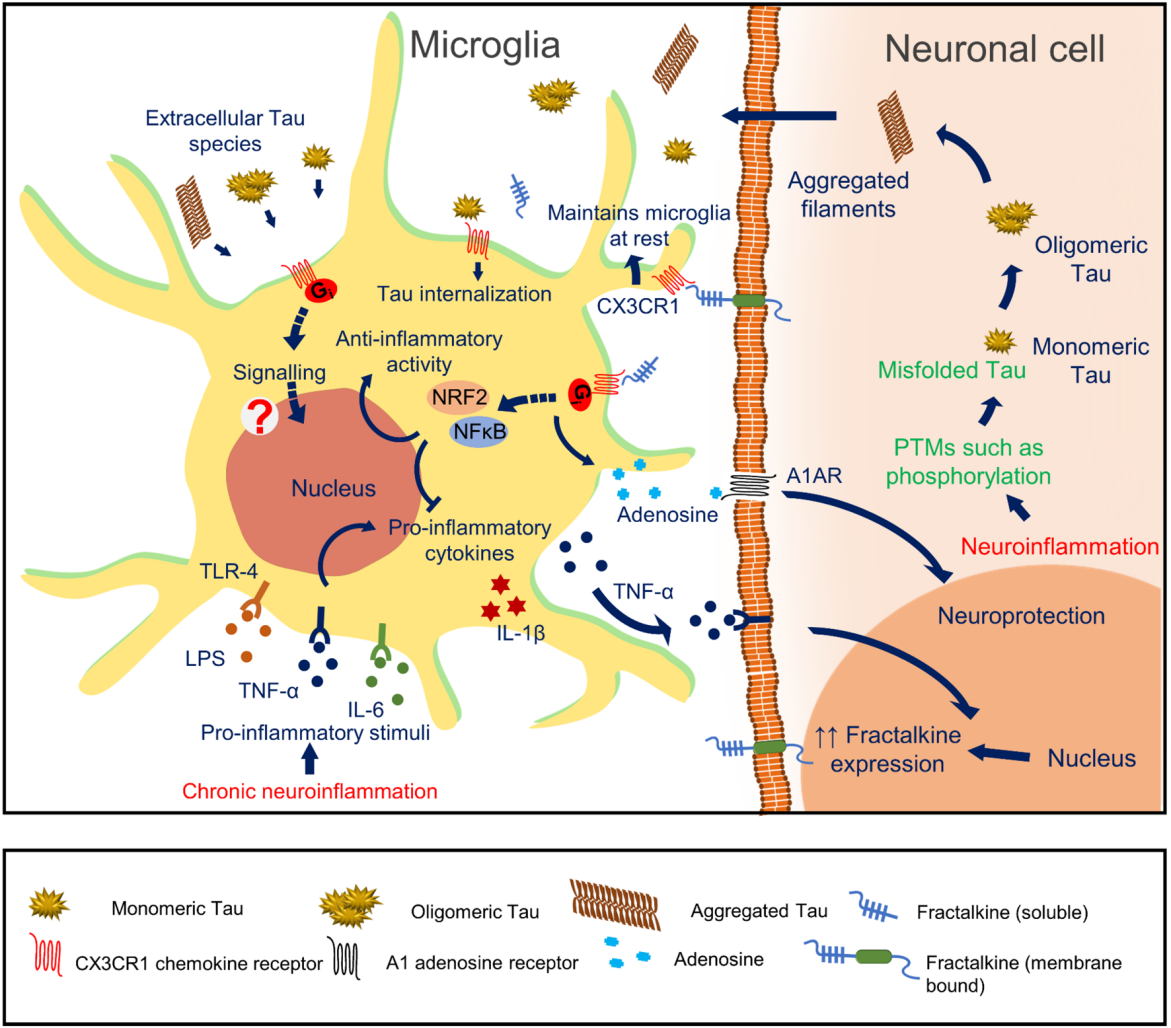
CX3CR1/ Fractalkine signalling—different roles in Microglia: In Alzheimer’s disease, chronic neuroinflammation increases the pro-inflammatory stimuli such as LPS, TNF-α and IL-6 in the CNS. Microglial cells, which are maintained at the resting stage under normal conditions, are activated by these pro-inflammatory stimuli by interacting through several surface receptors. Upon pro-inflammatory activation, microglia release excess of pro-inflammatory cytokines in the environment to activate more microglial cells to target the site of inflammation. Pro-inflammatory cytokine, IL-1β secreted from inflammatory microglia activates the TLR4 signalling pathway of neuronal cells and is involved in hyperphosphorylation of Tau protein by activating p38 MAP kinases. Fractalkine (CX3CL1), constitutively expressed neuronal protein, is either present as soluble or membrane-bound maintains the microglia in a quiescent stage. The pro-inflammatory TNF-α secretion by the microglial cells activates TNF receptors of neuronal cells and promotes the expression of fractalkine. Fractalkine interacts with CX3CR1 and activates G_i_ protein-mediated signalling pathway that activates nuclear transcription factors, NFκB and NRF2. CX3CR1/CX3CL1 activation also inhibits pro-inflammatory cytokine synthesis and microglial activation. In AD conditions, misfolded Tau aggregated to form oligomers and filaments that spread across the CNS. Also, the extracellular Tau interacts with CX3CR1 for its internalization

Fractalkine is involved in the inhibition of microglial activation through the CX3CR1 receptor by activating PI3K and Akt signals and maintains microglia in a quiescent state [87]. In aged rats, the level of fractalkine mRNA and fractalkine was reduced and observed with enhanced microglial activation, MHC II, CD40 mRNA levels and IL-1β secretion. Fractalkine treatment also reduced age-related increase in MHC II, CD40 mRNAs, IL-1β levels and reduced microglial activation in aged rats [87]. Fractalkine is reported to inhibit the release of TNF-α, a pro-inflammatory cytokine released by LPS activated microglia [88]. Moreover, IL-1β (pro-inflammatory cytokine) synthesis by LPS activated microglia, and its neuronal receptor TLR-4 signalling is also reduced on fractalkine exposure in PI3K and Akt dependent manner that denotes the anti-inflammatory effect of CX3CL1 (both soluble and membrane-bound form) [87]. Under pathological conditions, TLR-4 signalling is reported to be involved in Tau phosphorylation through p38 MAP kinases that are regulated by CX3CL1 signalling [89]. In 2014, Becker et al*.* demonstrated that fractalkine treatment activates nuclear transcription factor such as nuclear factor erythroid 2-related factor 2 (NRF2) mediated by PI3K and Akt activation, GSK-3β inactivation in microglial cells [90]. These nuclear transcription factors are involved in the synthesis of antioxidant enzymes, including *heme oxygenase 1* and attenuate the pro-inflammatory phenotype in microglia [90]. In 2018, controversial results were reported by the same group that CX3CR1 receptor signalling is involved in the synthesis of pro-inflammatory cytokines such as IL-1β, TNF-α, etc*.,* mediated by MSK-1 and NFκB [91]. CX3CR1 signalling also triggered kinases such as ERK-1/2, p38 and JNK in immortal microglial (IMG) cells [91]. In addition, CX3CR1 deficient primary microglia had been observed with impaired NRF2, deficiency of phagocytosis and impaired microglial migration [92] (Fig. 1).

## CX3CR1/Fractalkine in neurons—role in neuroprotection, neurogenesis, learning, memory and synaptic plasticity

**Fig. 2:**
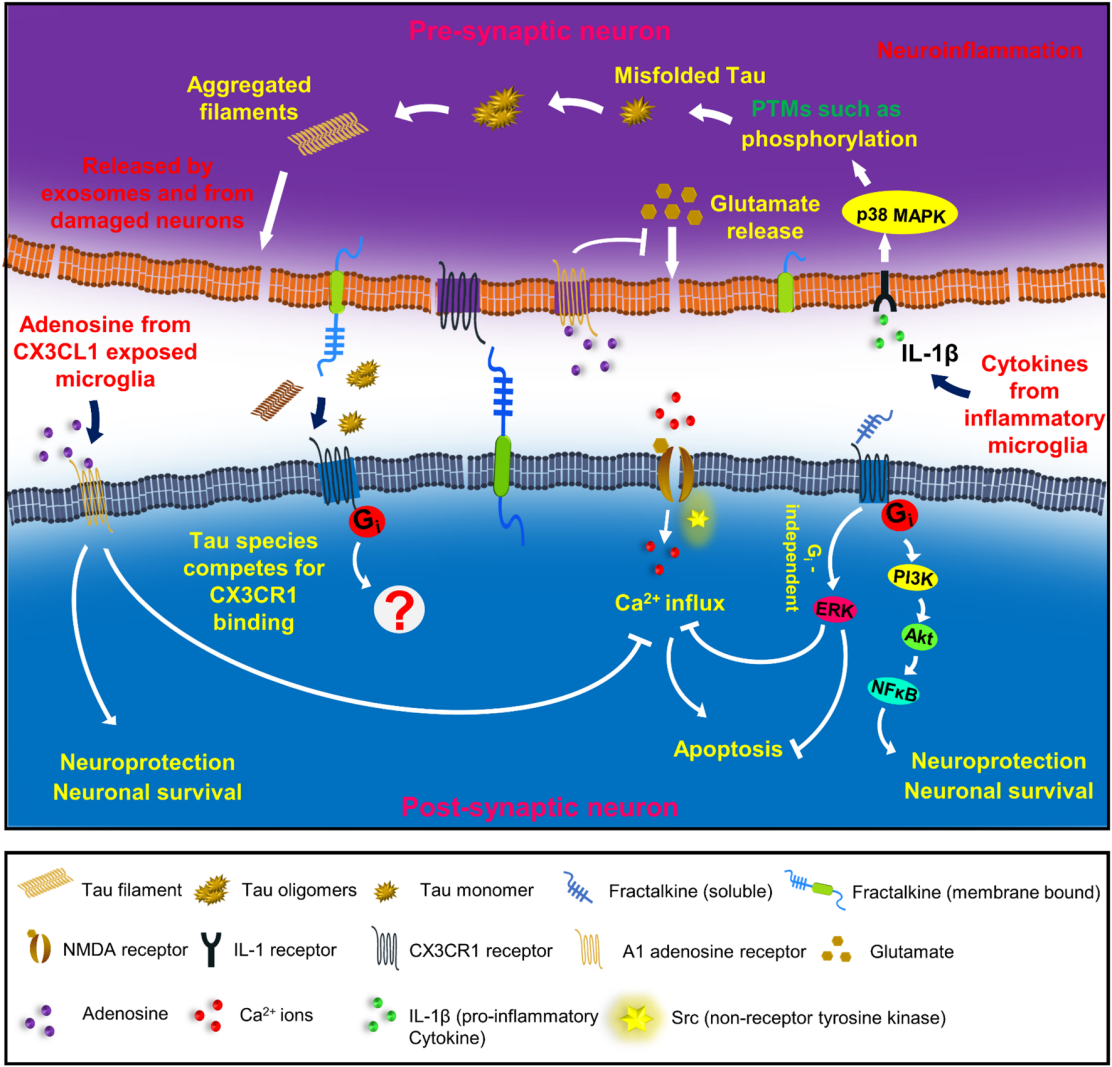
CX3CR1/ Fractalkine signalling—pathophysiological roles in neurons: In neuronal cells, fractalkine plays a significant role in neuroprotection, neurogenesis, learning, memory and synaptic plasticity. The chemokine receptor is involved in G_i_-protein-mediated signalling that leads to the activation of the transcription factor, NFκB mediated by PI3K and Akt signals. NFκB is involved in neuronal survival and neuroprotective effects. Fractalkine is also involved in Gi-protein independent activation of ERK kinases that inhibits NMDA receptor-mediated calcium influx and neuronal apoptosis. Fractalkine exposed microglia releases adenosine that is involved in neurotrophic effects via A1 adenosine signalling mechanisms. In neurodegenerative diseases, neurons undergo chronic inflammation, and Tau forms oligomers and filaments due to various post-translational modifications such as phosphorylation. Inflammatory microglia release several pro-inflammatory cytokines such as IL-1β, TNF-α and IL-6. IL-1β promotes Tau phosphorylation and aggregation mediated by IL-1 and TLR-4 receptors. Extracellular Tau interacts with CX3CR1 receptor in microglia for its internalization. We hypothesize that extracellular Tau species may also have possible interaction with neuronal receptor and differential signalling towards neuronal inflammation and neurodegeneration

Apart from the indirect effects of fractalkine on neuronal cells through microglial signalling, there are several direct roles of fractalkine in neuronal cells. In the CNS, fractalkine is involved in anti-inflammatory effects and protects neuronal cells against neuroinflammation and tissue damage. Fractalkine treatment to neuronal cells led to the activation of ERK and Akt kinases upon transient phosphorylation [93]. Fractalkine also has its role on other receptors such as NMDA receptors, Adenosine receptors, etc., It inhibits NMDA receptor-induced calcium influx and apoptosis; activates the transcription factors such as CREB and NFκB; ultimately involved in learning, memory and synaptic plasticity-mediated by ERK activation [93]. It promotes the release of adenosine from activated microglia, which in turn activates A1 adenosine receptor that is involved in neuroprotection [94]. Fractalkine is also involved in hippocampal neurogenesis that is involved in learning and memory. It also acts as neuroprotectant and protects the neuron from neuroinflammation and neuronal injury. Fractalkine expression in aged rats has been reduced, which led to increased CX3CR1 expression and microglial activation [95]. Similar conditions occur in advanced conditions of AD, the expression of fractalkine reduces that increases microglial activation ultimately leading to neuronal inflammation, loss of synapses and cognitive deficits (Fig. 2).

## CX3CR1 knockout studies in AD models—role in Aβ and Tau clearance

Microglial CX3CR1/CX3CL1 axis plays a significant role in the progression of Alzheimer’s disease with controversy in Aβ and Tau pathology [96]. CX3CR1 impairment reduces Aβ-mediated pathology and promotes Aβ clearance. In hTau/CX3CR1^−/−^ mice models, microglial activation by lipopolysaccharides promoted p38 MAPK-mediated Tau hyperphosphorylation and behavioural impairments. Further, it is proved that microglial activation led to secretion of pro-inflammatory cytokine IL-1 that promotes Tau hyperphosphorylation and aggregation by TLR4-mediated pathway in neurons [89]. In hAPP mice models, CX3CR1 knockout promoted Tau pathology (hyperphosphorylation) and cognitive deficits with no significant changes in APP processing and levels of Aβ [97], whereas Lee et al., demonstrated that CX3CR1 deficiency regulates Aβ deposition, promotes microglial phagocytosis and Aβ clearance in APPPS-1 and R1.40 mice models [98]. Further, Aβ significantly reduced CX3CR1 expression and impaired CX3CR1 signalling in cultured microglial cells and AD brain [97].

## CX3CR1/Tau signalling—role in pro-inflammatory cytokine synthesis, microglial activation and migration

In AD brain, neurons undergo severe inflammation and tissue damage that spreads aggregated Tau species such as oligomers and filaments around the microglial environment. Bolos et al*.* demonstrated that extracellular Tau is capable of binding to N-terminal peptides of CX3CR1 and promotes its internalization in microglial cells [40]. In AD brain, the expression level of fractalkine is reduced, and hence Tau competes to interact with this receptor. Further, CX3CR1/CX3CL1 signalling is involved in the synthesis of anti-inflammatory cytokines and also has a significant role in cytoskeletal rearrangement, migration, apoptosis and proliferation. On Tau exposure to microglial cells, the p38 pathway gets altered and activated with a significant change in the pro-inflammatory cytokine expression profile [72]. Pro-inflammatory cytokines such as Il-6, IL-1β, TNF-α and Mip-1α has been synthesized by these microglial cells [72]. Similarly, in AD, highly accumulated extracellular Tau interacts with the chemokine receptor, CX3CR1 and prevents CX3CL1 from binding to its receptor. We hypothesize that CX3CR1/Tau signalling could be involved in the synthesis of pro-inflammatory cytokines, enhanced microglial activation and migration that ultimately leads to neuro-inflammation and neuronal damage in neurodegenerative diseases such as Alzheimer’s disease.

## Conclusion

Microglia plays a critical role in the progression of Alzheimer’s disease. Microglia are capable of internalizing and degrading both Aβ deposits and Tau aggregates [14, 35, 40]. In AD, neuroinflammation leads to excess activation of microglia promoting migration, cell proliferation and impaired phagocytosis. The role of extracellular Tau species in CX3CR1 receptor interaction, signalling, inflammatory activation and proliferation of microglia in Alzheimer’s disease is still poorly understood and further needs to be addressed. This study would help to know the mechanism of CX3CR1/Tau signalling in impaired phagocytosis, enhanced migration, proliferation and neuroinflammation.

## Data Availability

Not applicable.

## Abbreviations

AD: Alzheimer’s disease
Aβ: Amyloid-β
CX3CL1: Fractalkine
CNS: Central nervous system
APP: Amyloid precursor protein
sAPPα: Soluble amyloid precursor protein-α
AICD: APP intracellular domain
GPCRs: G-protein coupled receptors
PRD: Proline-rich domain
GRKs: G-protein coupled receptor kinases
GSK-3β: Glycogen synthase kinase-3β
ERKs: Extracellular signal-regulated kinases
CDK5: Cyclin-dependent kinase-5
ADAM10: A disintegrin and metalloproteinase domain-containing protein 10
ADAM17: A disintegrin and metalloproteinase domain-containing protein 17
MSK-1: Mitogen- and stress-activated kinase-1
TLR-4: Toll-like receptor-4
TREM-2: Triggering receptor expressed on myeloid cells 2
NRF-2: Nuclear factor erythroid 2 (NFE2)-related factor 2
CREB: CAMP responsive element binding protein
NFκB: Nuclear factor kappa-light-chain-enhancer of activated B cells
PI3K: Phosphoinositol-3 kinase
Akt: Protein kinase-B
IL-1β: Interleukin-1β
IL-6: Interleukin-6
IFN-γ: Interferon-γ
TNF-α: Tumour necrosis factor-α
Mip-1α: Macrophage inflammatory protein 1-alpha
CD200: Cluster of differentiation 200
CD40: Cluster of differentiation 40
MHC: Major histocompatibility complex
